# *Malassezia pachydermatis* from brown bear: A comprehensive analysis reveals novel genotypes and distribution of all detected variants in domestic and wild animals

**DOI:** 10.3389/fmicb.2023.1151107

**Published:** 2023-04-12

**Authors:** Suzana Hađina, Branka Bruvo Mađarić, Snježana Kazazić, Tina Paradžik, Slaven Reljić, Ljiljana Pinter, Đuro Huber, Dušica Vujaklija

**Affiliations:** ^1^Department of Microbiology and Infectious Diseases with Clinic, Faculty of Veterinary Medicine, University of Zagreb, Zagreb, Croatia; ^2^Division of Molecular Biology, Ruđer Bošković Institute, Zagreb, Croatia; ^3^Division of Physical Chemistry, Ruđer Bošković Institute, Zagreb, Croatia; ^4^Department of Forensic and State Veterinary Medicine, Faculty of Veterinary Medicine, University of Zagreb, Zagreb, Croatia; ^5^Department of Biology, Faculty of Veterinary Medicine, University of Zagreb, Zagreb, Croatia

**Keywords:** novel genotypes, brown bear, *Ursus arctos*, MALDI-TOF mass spectra, *Malassezia pachydermatis*, multilocus genotyping

## Abstract

*Malassezia pachydermatis* (phylum *Basidiomycota*, class *Malasseziomycetes*) is a zoophilic opportunistic pathogen with recognized potential for invasive infections in humans. Although this pathogenic yeast is widespread in nature, it has been primarily studied in domestic animals, so available data on its genotypes in the wild are limited. In this study, 80 yeast isolates recovered from 42 brown bears (*Ursus arctos*) were identified as *M. pachydermatis* by a culture-based approach. MALDI-TOF mass spectrometry (MS) was used to endorse conventional identification. The majority of samples exhibited a high score fluctuation, with 42.5% of isolates generating the best scores in the range confident only for genus identification. However, the use of young biomass significantly improved the identification of *M. pachydermatis* at the species confidence level (98.8%). Importantly, the same MALDI-TOF MS efficiency would be achieved regardless of colony age if the cut-off value was lowered to ≥1.7. Genotyping of LSU, ITS1, CHS2, and β-tubulin markers identified four distinct genotypes in *M*. *pachydermatis* isolates. The most prevalent among them was the genotype previously found in dogs, indicating its transmission potential and adaptation to distantly related hosts. The other three genotypes are described for the first time in this study. However, only one of the genotypes consisted of all four loci with bear-specific sequences, indicating the formation of a strain specifically adapted to brown bears. Finally, we evaluated the specificity of the spectral profiles of the detected genotypes. MALDI-TOF MS exhibited great potential to detect subtle differences between all *M. pachydermatis* isolates and revealed distinct spectral profiles of bear-specific genotypes.

## Introduction

The genus *Malassezia* currently includes 18 species of lipophilic basidiomycetous yeasts (Lorch et al., 2018; Theelen et al., 2018). Although various metagenomic studies have demonstrated the occurrence of *Malassezia* phylotypes in diverse ecological niches (Amend, 2014), most of them are reported to colonize or infect the skin and mucosa of humans or animals. Thus, *Malassezia* spp. are generally considered opportunistic pathogens, some species of which exhibit host specificity (Cabanes, 2014; Velegraki et al., 2015). Under normal physiological conditions, this yeast lives in equilibrium with other members of the skin microbiota. Moreover, in adult humans, *Malassezia* is the dominant fungal genus (Byrd et al., 2018) accounting for 53–80% of the total yeast population on healthy skin (Gao et al., 2010). However, this biological balance can be disturbed by various factors, leading to yeast overgrowth associated with various clinically manifested infections (Gaitanis et al., 2012; Velegraki et al., 2015; Prohic et al., 2016).

Due to its zoonotic potential, *Malassezia pachydermatis* has attracted considerable attention (Chang et al., 1998; Morris, 2005). This species has been recognized as a causative agent of fungemia, predominantly in immunocompromised patients (Lautenbach et al., 1998; Roman et al., 2016; Lee et al., 2019), children, and neonates (Chryssanthou et al., 2001; Al-Sweih et al., 2014; Ilahi et al., 2018; Chow et al., 2020; Teoh et al., 2022). It has been predicted that systemic infections caused by *M. pachydermatis* may be underdiagnosed by standard diagnostic procedures (Cabanes, 2014). In this context, rapid and correct identification of this yeast is essential for appropriate life-saving medical treatment (Kolecka et al., 2014).

In domestic animals, *M. pachydermatis* has been isolated mainly from dogs and cats (Guillot and Bond, 2020), but it has also been found in pigs, horses, goats, and other animals (Pinter et al., 2002; Sugita et al., 2010; Eguchi-Coe et al., 2011; Shokri, 2016). Early studies also reported the presence of *M. pachydermatis* in captive wild animals (Sugita et al., 2010). Although recent studies have shed some light on commensalism, pathogenicity and genetic variability of *M. pachydermatis* and other members of this genus in the wild (Dall’ Acqua Coutinho et al., 2006; Gandra et al., 2008; Lorch et al., 2018; Puig et al., 2018; Coutinho et al., 2020), available data for genotypes found in the wild are still very limited.

Various molecular methods are used to systematically identify *Malassezia* species directly from skin samples (Sugita et al., 2010). Genomic markers, such as LSU, ITS, IGS, CHS2, or β-tubulin, are used for epidemiological or phylogenetic analyses (Cafarchia et al., 2007; Castella et al., 2014; Cho and Sugita, 2016). Previously reported data show high genetic diversity among the *Malassezia* species and various degrees of intraspecific variability (Makimura et al., 2000; Gupta et al., 2004). For *M. pachydermatis*, 15 distinctive genotypes have been found in domestic animals (Puig et al., 2016, 2017). In addition to DNA analyses, protein profiling by matrix-assisted laser desorption/ionization time-of-flight (MALDI-TOF) mass spectrometry (MS) is increasingly becoming the technique of choice for routine yeast identification (Patel, 2019; Robert et al., 2021).

Considering the scarcity of data on *Malassezia* populations in the wild, the objectives of this research were (i) to investigate the occurrence of *Malassezia* species in brown bears inhabiting the mountainous region of Croatia, (ii) to evaluate the performance of MALDI-TOF MS for reliable identification of isolates from the wild, and (iii) to characterize population genetic diversity using a combination of selected DNA markers. These objectives allowed us to identify previously unreported bear-specific genetic variants of *M. pachydermatis* and to define their mass spectrum profiles. The results obtained in this study indicate the adaptive capacity of this commensal yeast to inhabit a wide host range, thus highlighting the possible natural reservoirs for its transmission potential between wild and domestic animals.

## Materials and methods

### Sample collection

**Figure 1:**
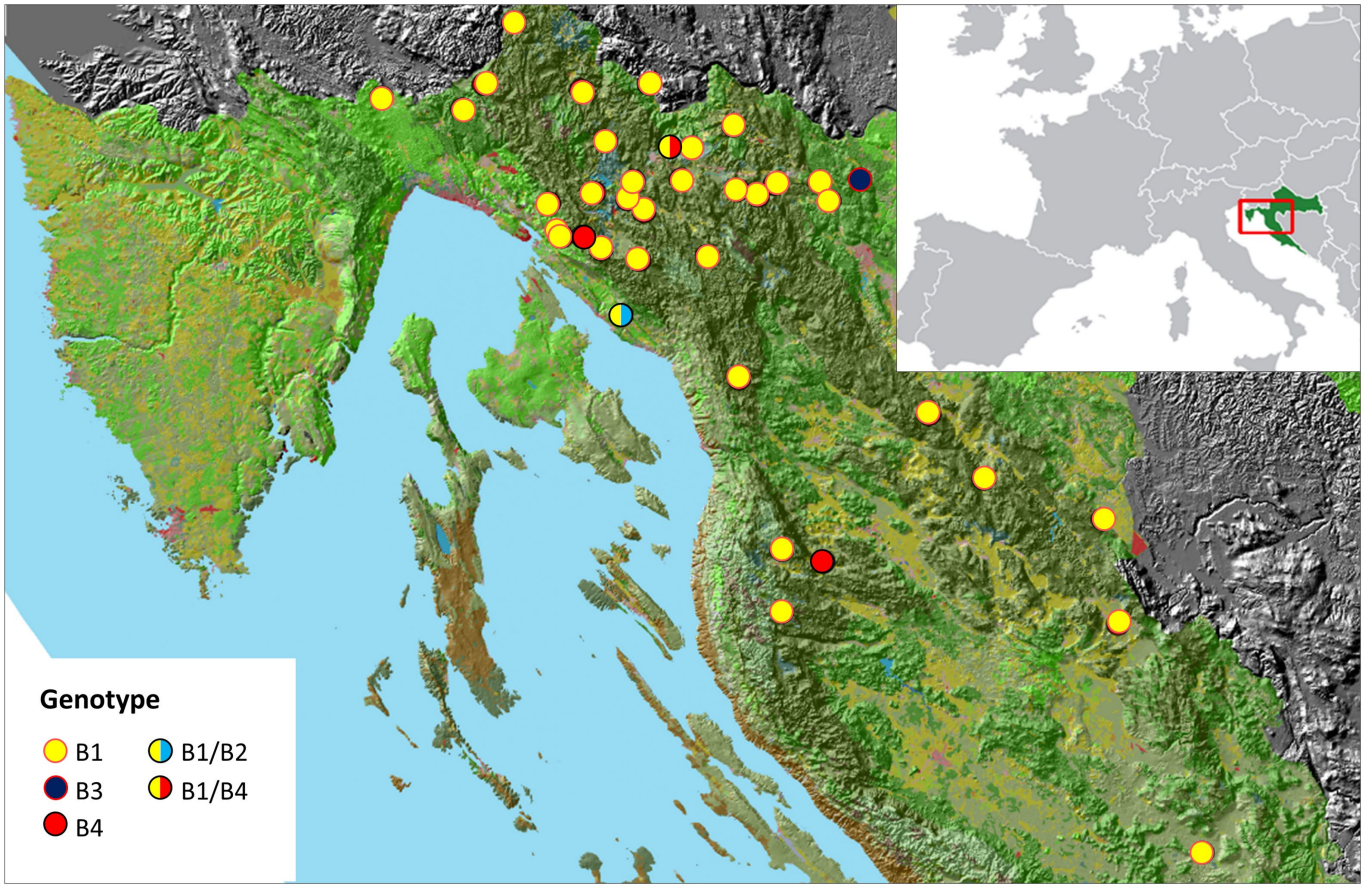
A map of the sampling sites in a mountainous region of Croatia. *Malassezia pachydermatis* genotypes identified in this study are shown in legend.

A total of 129 samples were collected from 42 brown bears, using sterile swabs to sample the left/right external ear canal and anus. Samples were collected within 12 h *post mortem* of animals after various accidents or during anesthesia from live animals captured for telemetry studies (Project: Life Dinalp Bear, Life13 Nat/Si/000550) in accordance with the permits issued by the Committee of Veterinary Ethics of the Faculty of Veterinary Medicine, University of Zagreb, the Ministry of Agriculture and the Ministry of Environmental and Nature Protection, Republic of Croatia. Geospatial mapping was performed using ArcGIS software v.10.2 (Redlands, California, United States). Sampling sites are shown in Figure 1, while location coordinates of bears are listed in Supplementary Table S1.

### Isolation, cultivation, and identification of *Malassezia* isolates

Swabs were plated on modified Dixon agar (36 g malt extract, 10 g peptone, 20 g desiccated ox-bile, 10 mL Tween 40, 2 mL glycerol, 2 mL oleic acid, and 15 g agar per liter, pH adjusted to 6.0) recommended for isolation and differentiation of *Malassezia* species (Guého-Kellermann et al., 2010). The agar was supplemented with 0.5% chloramphenicol and 0.5% cycloheximide, to prevent the growth of bacteria and molds, respectively. The plates were incubated at 32° C for 14 days. Isolates were identified using routine laboratory diagnostic methods, as follows. All isolates that showed the appearance of colonies typical for *Malassezia*, a yellowish-creamy and smooth or lightly wrinkled surface with a buttery consistency, were selected (Guého-Kellermann et al., 2010). Smears from the selected colonies were stained with the Bio-Diff RTU kit (Biognost, Croatia) and examined under the microscope for “bottle-shaped” morphology that characterizes *Malassezia* cells. Subsequently, the lipid dependence for the growth of *Malassezia* isolates was determined on Sabouraud glucose (SGA) agar. After phenotypic identification, isolates were stored at −80° C until further testing.

#### MALDI-TOF MS analysis

For the MALDI-TOF MS analyses, direct on-plate extraction and in tube full protein extraction method were applied, as recommended by the manufacturer (Bruker Daltonik, Germany). For protein extraction, a yeast biomass (~2 μL volume) grown on SGA agar for up to 3 days was collected with a pipette tip, suspended in 300 μL HPLC-grade water (Sigma Aldrich) and mixed thoroughly. Nine hundred microliter of absolute ethanol was added to the suspension and mixed well. The samples were then centrifuged at 13,000 rpm for 2 min, the supernatant was removed, and the pellets were air dried at room temperature (RT). The pellets were re-suspended in 70% formic acid to disrupt the cell wall. The volume of formic acid was adjusted according to the pellet size (1:1 by volume). An equal volume of acetonitrile was added, and the solution was mixed by vortexing for 1 min and centrifuged at 13,000 rpm for 2 min. One microliter of this supernatant was placed on a 96-spot polished steel target plate and air dried at RT. Each sample was overlaid with 1 μL of saturated α-cyano-4-hydroxycinnamic acid in 50% acetonitrile and 2.5% trifluoroacetic acid and air dried at RT.

MS measurements were performed using a Microflex LT MALDI-TOF mass spectrometer and FlexControl 3.0 software (Bruker Daltonics, Germany) for automatic acquisition of mass spectra. Each mass spectrum was generated with 240 laser shots from the same spot in six different positions while each isolate was processed in triplicate and in at least two spots. External calibration was performed using the Bruker Bacterial Test Standard. The acquired mass spectra were processed with the MALDI Biotyper 3.1 software package using the default settings. MALDI Biotyper scores for identification were expressed as log(score) values. Scores ≥2 are indicative of reliable species identification, from 1.7 to 1.999 as reliable genus identification, and scores <1.7 as unreliable identification. A score-oriented dendrogram of MALDI-TOF mass spectra profiles (MSP) was generated using MALDI Biotyper 3.0 software with the following settings: distance measure was set to Euclidian and linkage to average.

### DNA extraction, PCR amplification, and sequencing of ITS1, LSU, CHS2, and β-tubulin DNA regions

A loopful of yeast biomass (up to 30 mg) was harvested from a culture grown on SGA for 2 to 3 days. Genomic DNA was extracted using PureLink Genomic DNA Mini Kit (Invitrogen, United States) according to the protocol described by the manufacturer. DNA concentration was validated spectrophotometrically using the BioDrop μLITE (BioDrop, United Kingdom), and the quality of each DNA sample was assessed by standard agarose gel electrophoresis. The purified DNA samples were stored at −80°C until use.

DNA regions encoding the large ribosomal subunit (LSU), internal transcribed spacer 1 (ITS1), chitin synthase 2 (CHS2), and β-tubulin were amplified from 80 *M. pachydermatis* DNA samples using previously described primers (Supplementary Table S2) and PCR protocols (Fell et al., 2000; Makimura et al., 2000; Cafarchia et al., 2007; Castella et al., 2011). Amplified DNA fragments were checked on 1% agarose gels, and enzymatically purified using Exonuclease I and Thermosensitive Alkaline Phosphatase (Thermo Scientific), according to manufacturer’s protocol. The purified PCR products were submitted to Macrogen Europe for sequencing. DNA fragments were sequenced from both directions using the same primers as in the PCR reactions. Sequence chromatograms were visually inspected, and the sequences were assembled in Geneious 8.1.4 (Kearse et al., 2012) and BioEdit 7.2.5. programs (Hall, 1999). The CHS2 and β-tubulin gene fragments were translated into protein sequences in order to confirm the continuity of the open reading frames. The sequences were deposited in NCBI GenBank under the accession numbers listed in Table 1.

**Table 1:** Distribution of gene locus variants (A) LSU, ITS1, CHS2 and β-tubulin sequence types from *M. pachydermatis* isolates identified in brown bears (this study), with NCBI accession numbers; (B) Representative sequences of gene locus variants published previously.

A	LSU sequence types	ITS1 sequence types	CHS2 sequence types	β-tubulin sequence types
*M. pachydermatis* sequence variants reported in this study	**LSU-BI (OQ519274)** All isolates except 1, 2, 29; identical to *LSU-I* (see Table 1B)	**ITS1-BI (OQ519261)** All isolates except 1, 2, 29; identical to *ITS1-I* (see Table 1B)	**CHS2-BI (OQ550025)** All isolates except 1,2, 24, 29; identical to *CHS2-I* (see Table 1B)	**β-tub-BI (OQ550028)** All isolates except 1,2, 24, 29, 68; identical to *KC573803* (see Table 1B)
			**CHS2-BII**^1^ **(OQ550026)** Isolate 24; identical to *CHS2-II* (see Table 1B)	**β-tub-BII**^2^^,^ ^3^**(OQ550029)** Isolates 24&68; novel sequence
	**LSU-BII**^3^ **(OQ519275)** Isolates 1, 2, 29; novel sequence	**ITS1-BII**^3^ **(OQ519262)** Isolates 1, 2, 29; novel sequence	**CHS2-BIII**^3^ **(OQ550027)** Isolates 1, 2, 29; novel sequence	**β-tub-BIII**^3^ **(OQ550030)** Isolates 1, 2, 29; novel sequence
B				
*M. pachydermatis* previously reported sequence variants^4^	**AY743605–dog, horse, cat (LSU-I)** KU313705–goat, pig (LSU-II) KU313706–dog (LSU-III) KU313707–cat (LSU-IV) KU313708–dog (LSU-V)	**AY743637–dog (ITS1-I)** DQ915505–dog EU158829–dog HG529981–human pre-term neonate KU313709–dog (ITS1-II) KU313710–horse (ITS1-III) KU313711–goat, dog (ITS1-IV) KU313712–cat (ITS1-V) KU313713–dog (ITS1-VI) KU313714–dog (ITS1-VII) KU313715–cat (ITS1-VIII) KU313716–pig (ITS1-IX) KU313717–cat (ITS1-X) KU313718–dog (ITS1-XI) KY655274–dog (ITS1-XII) KY655275–dog (ITS1-XIII)	**EF140657–dog, horse (CHS2-I)** **KU313719–dog (CHS2-II)** KU313720–dog (CHS2-III) KU313721–goat, dog (CHS2-VI) KU313722–cat (CHS2-V) KU313723–cat (CHS2-VI) KU313724–dog (CHS2-VII) KU313725–pig (CHS2-VIII) KU313726–dog (CHS2-IX)	**KC573803–dog, horse (β-tub -I)** KU313727–dog, horse (β-tub -II) KU313728–goat, dog (β-tub -III) KU313729–cat (β-tub -IV) KU313730–cat (β-tub -V) KU313731–dog (β-tub -VI) KU313732–pig (β-tub -VII) KU313733–dog, cow (β-tub -VIII) KY655276–dog (β-tub -IX

1 1nt difference with respect to CHS2-BI.

2 1nt difference with respect to β-tub-BI.

3 Not previously reported.

4 Sequence variants within the respective gene loci. Each sequence represents previously reported gene locus type (Cabañes et al., 2005, 2007; Castella et al., 2014; Puig et al., 2016).

### Multiple sequence alignment and phylogenetic analysis

Haplotypes of ITS1, LSU, CHS2, and β-tubulin DNA sequences of *M. pachydermatis* isolates were aligned in the online version of MAFFT v. 7 (Katoh and Standley, 2013) under default parameters, together with the corresponding gene sequences of *M. pachydermatis* from domestic animals available in the public database NCBI (Cabañes et al., 2005; Puig et al., 2016, 2017). Sequences of the corresponding molecular markers from two other congeneric species (*Malassezia furfur*, Acc. numbers HM177260, EU513202, KC573799; *M. sympodialis*, Acc. numbers AY743628, AY743657, XM_018885767, KC573797) were used as outgroups (Cabañes et al., 2005; Ran et al., 2008; Cafarchia et al., 2011; Castella et al., 2014; Puig et al., 2018). In addition, a combined dataset corresponding to distinct genotypes of *M. pachydermatis* from bears was constructed in BioEdit 7.2.5. (Hall, 1999) through concatenation of respective sequence haplotypes of four molecular markers (LSU, ITS1, CHS2, and β-tubulin). The genotypes of 19 strains defined previously (Puig et al., 2017) were also included in the combined dataset.

Five datasets (LSU, ITS1, CHS2, β-tubulin, and concatenated alignment; available in TreeBase^1^) were used in subsequent phylogenetic analyses. For each separate partition, uncorrected p-distances were calculated in Mega 7 (Kumar et al., 2016). Neighbor joining (NJ) phylogenetic trees were constructed in Mega 7 based on p-distances matrix, with pairwise deletion of gap positions. The best-fit nucleotide substitution models were determined for each separate partition under the Akaike information criterion in Jmodeltest 2.0 (Darriba et al., 2012) (K2 model for the LSU dataset, JC model for the ITS1 dataset, K2 + G for the CHS2 dataset and T92 + G for the β-tubulin dataset). Maximum likelihood (ML) aLRT analyses for separate partitions were performed online in PhyML 3.0^2^ (Guindon et al., 2010) under best-fit models. The concatenated matrix was analyzed in PhyML using the “Automatic model selection by SMS” option (Lefort et al., 2017) and the Akaike information criterion. Branch support values were estimated from 1,000 bootstrap replicates in NJ and by aLRT in ML analyses. Phylogenetic trees in .nwk format are available in TreeBase.

## Results and discussion

### Isolation of *Malassezia pachydermatis* from brown bears

*M. pachydermatis* has been isolated mainly from dogs but it has also been found in other domestic animals (Theelen et al., 2018; Guillot and Bond, 2020) and humans (Gao et al., 2010). However, little is known about the phylotypes and pathogenicity of *M. pachydermatis* in wild habitats. Since the relatedness between yeast genotypes and host species has already been reported (Cabanes, 2014; Velegraki et al., 2015), we envisioned that a comparative study of the genetic variants of *M. pachydermatis* from broader ecological niches might provide deeper insight into its potential to colonize diverse hosts, and thus provide the basis for studying its zoonotic potential. Although the occurrence of *M. pachydermatis* in bears and other wild animals was detected several decades ago, as reviewed by Sugita et al. (2010), data on its genetic variability are lacking. In Croatia, the brown bear inhabits the mountains of Gorski Kotar and Lika in an area of about 9,600 km2 (Huber et al., 2008), which provides an opportunity to collect and analyze yeast isolates from distant locations (Figure 1; Supplementary Table S1). As expected, the selective medium that was used mainly grew colonies that resembled *Malassezia* sp. by their morphology. Noteworthy, we occasionally noticed the growth of *Candida* sp. but their numbers were negligible and they were not further analyzed. All selected isolates exhibited non-lipid dependent growth so they were identified as belonging to the species *M. pachydermatis*. Altogether, 80 yeast isolates (62%) were obtained out of 129 swab samples from 42 individuals. No significant difference in recovery was found when samples were taken from different skin sites.

Standard culture-based identification methods and biochemical characterization are not always definitive in the identification of *Malassezia* species and particularly in discriminating very close species or subspecies (Robert et al., 2021). For example, the recent discovery of peculiar lipid-dependent strains of *M. pachydermatis* demonstrates broad variability within this species, which includes rare atypical strains with special growth requirements (Puig et al., 2017). In the last ten years, methods based on MALDI-TOF MS have been increasingly applied for the identification of pathogenic microorganisms in clinical microbiology laboratories (Singhal et al., 2015). Because this method is very rapid and has been used for the identification of several *Malasezzia* species (Kolecka et al., 2014; Vlek et al., 2014; Honnavar et al., 2018), we used it to verify the identification of *M. pachydermatis* from bears obtained by conventional protocols and to investigate the performance of MALDI-TOF in yeasts isolated from the wild.

### MALDI-TOF MS identified yeast isolates from brown bears as *Malassezia pachydermatis*

**Figure 2:**
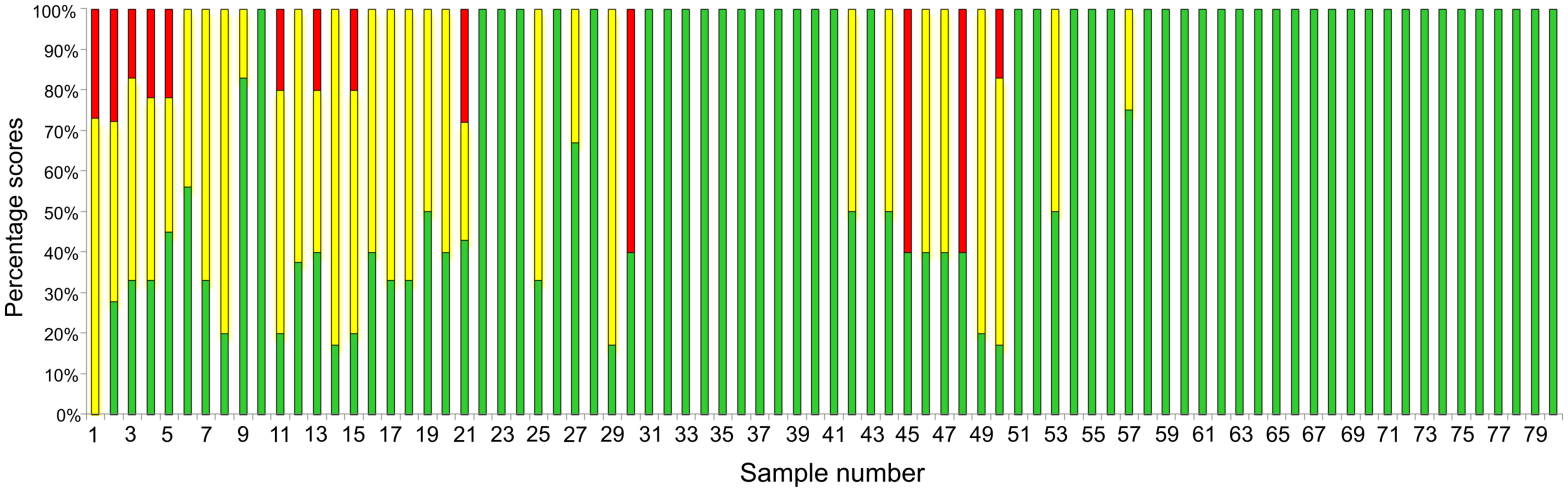
Presentation of MALDI-TOF MS sample scores. The sample number is shown on the *X*-axis while the proportion of the score ranges (percentage scores) obtained by MS analyses for each sample is shown on the *Y*-axis. Bars marked in green, yellow and red denote samples that showed greater fluctuation in score values (Supplementary Table S3). The use of young biomass (~48 h) yielded scores confident for species identification (≥ 2, green). The only exception was sample number 1, which only achieved a score confident for genus identification.

Initially, yeast isolates were prepared for MALDI-TOF MS using the direct on-plate extraction method. Due to the very poor quality of the protein spectra, this method was not applicable, so the full extraction protocol was applied to 80 isolates as described. Considering the MALDI Biotyper identification scores, it can be seen that a larger number of samples showed a significant fluctuation in the results. Of the 80 samples, MALDI-TOF MS correctly identified 46 samples (57.5%) as *M. pachydermatis* with a score ≥ 2 (2.007–2.412; green bars) (Figure 2). The other 34 isolates (42.5%) showed lower reproducibility between spots and yielded the best scores only in the confidence range for genus identification. The ranges of scores for all isolates are shown (Supplementary Table S3). The lower reproducibility between spots as well as the need to increase the number of runs to obtain a confident score has been reported previously for *Malassezia* species (Kolecka et al., 2014). We noticed that the colony texture of most isolates with lower scores appeared as if the colony was impregnated with grease and the biomass crumbled upon sampling. It has been reported that the texture of colonies can affect the quality of protein extracts (Denis et al., 2017). Therefore, 34 colonies were re-cultured and as soon as there was enough biomass (~ 48 h) MS analysis was performed. In this second round of identification, only one sample did not reach the species reliable score (≥2). The distribution of all scores obtained in the first and second runs was as follows: 21 isolates yielded scores 1.721–2.244 (yellow/green bars), while 9 isolates yielded scores 1.573–2.201 (red/yellow/green bars), 3 isolates yielded scores 1.422–2.249 (red/green bars), and only 1 isolate (red/yellow) never reached score 2 (1.556–1.956; red/yellow bar). To provide an overview of the fluctuation of scores, these data were used to create Figure 2. Our results clearly show that aged colonies significantly affected the range of scores, while using the biomass of young colonies improved score values to the species confidence level. This was particularly pronounced for three isolates (30, 45, and 48), which exhibited scores confident for species level only when young biomass was used. In contrast, a previous study reported that MS were reproducible for 2–5 days old *Malassezia* colonies (Denis et al., 2017). The discrepancy in the results could be attributed to the phenotypic characteristics of our isolates, which exhibited a different colony texture already after 3 days of growth, possibly due to more rapid aging of the colony. It should be pointed out, however, that all samples, even those that gave unreliable genus scores (red), scored *M. pachydermatis* as the first matching hit. It is important to note that in the last 10 years, numerous reports have suggested lowering the cutoff threshold (1.7–1.999) to improve the identification of different yeast species (Rosenvinge et al., 2013; Kolecka et al., 2014; Vlek et al., 2014; Robert et al., 2021). Consistent with this, by lowering the threshold score to ≥1.7, in our study MALDI-TOF MS would correctly identify 79/80 samples to species level (98.8%).

### Molecular genotyping revealed genetic diversity of *Malassezia pachydermatis* isolates from brown bears

Analyses of ribosomal genes and internal transcribed regions have been used primarily for phylogenetic characterization and genetic diversity analysis of different *Malassezia* species (Gemmer et al., 2002; Gupta et al., 2004; Gaitanis et al., 2006, 2009). An early study based on a single molecular marker, the 26S rRNA gene (Guillot et al., 1997), revealed seven genetic groups of *M. pachydermatis* in wild and domesticated carnivores, monkeys and humans. Further evaluation of genotype assignment using multiple genetic loci (CHS2, LSU, ITS1) identified three and four specific genotypes for *M. pachydermatis* isolated from domestic animals by Cafarchia et al. (2007) and Aizawa et al. (2001), respectively. The benefit of multilocus sequencing for differentiation between *Malassezia* genotypes has been further demonstrated in recent studies. Puig et al. (2017) reported the existence of 15 distinct genotypes as a result of sequence variations in four genomic loci (CHS2, LSU, ITS1, β-tubulin) of *M. pachydermatis*, also isolated from domestic animals. Using the same genetic loci, we examined the genetic variability of *M. pachydermatis* isolated from bears. PCR amplification resulted in ~640 bp LSU, ~280 bp ITS1, ~500 bp CHS2 and ~ 1,100 bp β-tubulin DNA products. Consistent with the results of the MALDI-TOF MS scores, all locus sequences exhibited the highest similarity to the *M. pachydermatis* gene loci deposited in the database. Analysis of these sequences revealed two distinct types for LSU and ITS1, and three for CHS2 and β-tubulin DNA regions, as shown in Table 1A. The majority of isolates have the sequence types LSU-BI, ITS1-BI, CHS2-BI, and β-tubulin-BI, which are identical to previously reported sequences from dogs, horses, and cats (Cabañes et al., 2005, 2007; Castella et al., 2014). Variations of these types (1 nt substitution) are observed for the CHS2 gene in one isolate (type CHS2-BII, previously reported) (Puig et al., 2016) and for the β-tubulin region in two isolates (type β-tubulin-BII, not previously reported). Novel sequence types for all markers analyzed, LSU-BII, ITS1-BII, CHS2-BIII and β-tub-BIII (Table 1A), were found in three isolates.

Distances between the sequence types of *M. pachydermatis* samples isolated from brown bears are low, accounting for only 1 nucleotide (nt) change (0.15%) in LSU, 1–4 nt changes (0.2–0.8%) in CHS2, 1–7 nt changes (0.1–0.6%) in β-tubulin, and 9 nt changes (3.2%) between different sequence types in the ITS1 gene region. The low variability in these genomic regions for *M. pachydermatis* isolated from bears is not surprising, as similar distance ranges were already observed for the same species isolated from other animals (Cafarchia et al., 2007; Puig et al., 2016). When comparing the sequence types (loci) of *M. pachydermatis* samples from bears with sequence types of *M. pachydermatis* samples isolated mainly from various domestic animals (Cabañes et al., 2007; Puig et al., 2016, 2017), the changes account for 0–7 nt (0–3.2%) in LSU, 0–12 nt (0–2.4%) in CHS2, 0–30 nt (0–2.7%) in the β-tubulin region, and 0–14 nt (0–5%) in ITS1. Overall, the observed differences are not very pronounced, suggesting that *M. pachydermatis* loci types from bears are most likely descended from a common ancestor after a recent transmission event, probably from dogs which exhibited the highest number of genotype variants to date. However, it is important to keep in mind that dogs represent the largest group studied and future studies may challenge this assumption.

**Figure 3:**
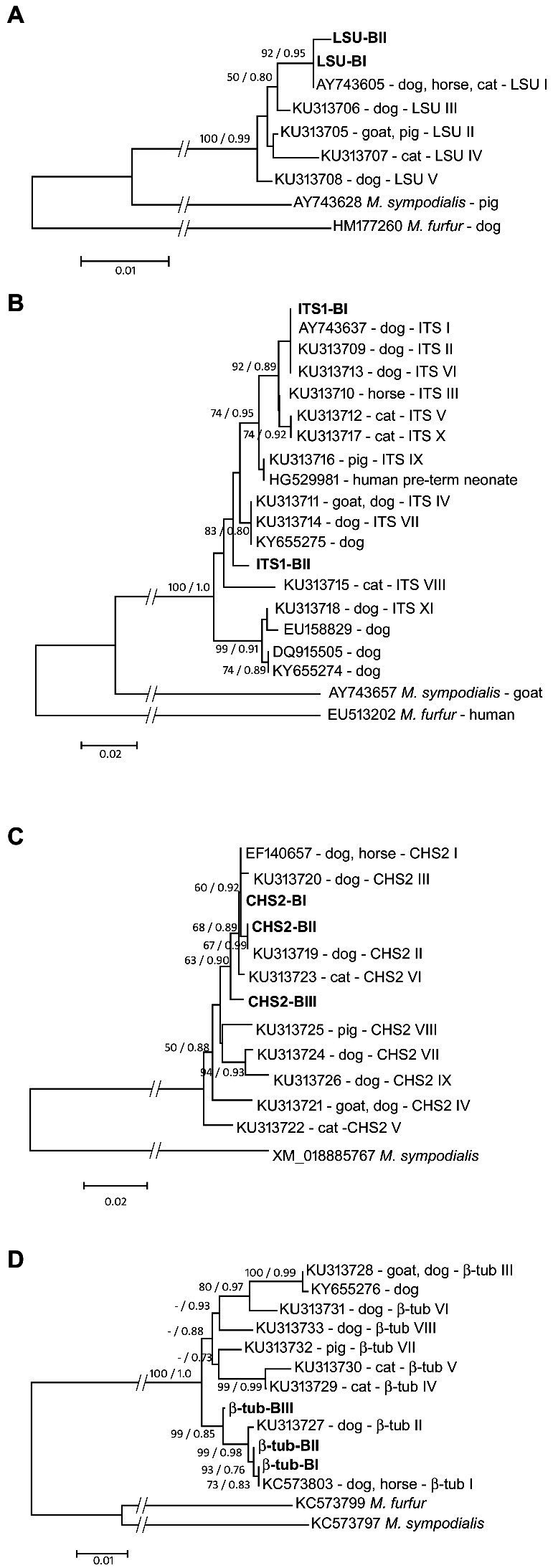
Maximum likelihood phylogenetic trees of *M. pachydermatis* gene loci [**(A)** LSU; **(B)** ITS1; **(C)** CHS2; **(D)** β-tub]. Reported gene types are labeled with accession numbers, host animal and respective gene type name. Gene types of *M. pachydermatis* isolates from brown bears are shown in bold. Node numbers denote NJ bootstrap support/ML aLRT support (values lower than 50%/0.70 are not shown). Sequences of the respective gene loci of *Malassezia furfur* and/or *Malassezia sympodialis* were used as outgroups.

The alignments of the LSU, CHS2 and β-tubulin sequences did not contain indels, whereas the ITS1 alignment contained multiple indel positions (alignments available in TreeBase). The final lengths of the alignments, including the previously published sequences of *M. pachydermatis* and of two outgroup species (*M. furfur* and *M. sympodialis*), were 254 bp for ITS1, 544 bp for LSU, 489 bp for CHS2, and 952 bp for the β-tubulin region. For each of the four molecular markers, phylogenetic analyses using neighbor joining and maximum likelihood methods resulted in identical tree topologies (Figures 3A–D).

As depicted in Figure 3A, two *M. pachydermatis* LSU sequence types found in bears are grouped in a strongly supported (92% bs – 0.95 aLRT) clade with the sequence AY743605 (Table 1B) found in dogs, horses, and cats (Puig et al., 2016). LSU-BI is identical to the sequence AY743605, whereas LSU-BII is found exclusively in bears and forms a distinct branch within this clade. The ITS1 sequences of *M. pachydermatis* found in the bear are grouped within one of the two major subclades in the phylogenetic tree, along with several sequence types reported for dogs, cats, and horses (Figure 3B). Type ITS1-BI is identical to type I found in dogs (AY743637; Table 1B), whereas type ITS1-BII forms a separate branch. Support for most of the nodes in this subclade is moderate or low. We further analyzed two protein-coding gene sequences, the chitin synthase (CHS2) and β-tubulin genes. As shown in Figure 3C, three *M. pachydermatis* CHS2 sequence types found in bears are grouped in a moderately supported (63% bs, 0.90 aLRT) clade with several sequence types found in dogs, horses and cats (Puig et al., 2016). The CHS2-BI and CHS2-BII types are identical to the EF140657 and KU313719 sequence types (Puig et al., 2016), respectively, as shown in Table 1B. The CHS2-BIII type, found in bears, has not been described previously and forms a separate branch within this clade. In Figure 3D, three β-tubulin sequence types of *M. pachydermatis* found in bears form a strongly supported clade (99% bs, 0.85 aRLT) with two previously reported β-tubulin sequence types (Puig et al., 2016). The β-tubulin-BI type is identical to KC573803 (Table 1B) found in dogs and horses, whereas the β-tubulin-BII and β-tubulin-BIII types, not previously reported in other animals, form separate branches.

The combinations of the described types of the four sequenced genomic regions of the respective *M. pachydermatis* isolates collected from bears resulted in four distinct genotypes, B1–B4. Each genotype comprises a specific combination of four loci sequence types: (i) Bear-B1 (75/80 isolates–LSU-BI/ITS1-BI/CHS2-BI/β-tub-BI), (ii) Bear-B2 (isolate 24–LSU-BI/ITS1-BI/CHS2-BII/β-tub-BII), (iii) Bear-B3 (isolate 68–LSU-BI/ITS1-BI/CHS2-BI/β-tub-BII), and (iv) Bear-B4 (isolates 1, 2, 29–LSU-BII/ITS1-BII/CHS2-BIII/β-tub-BIII).

The majority of *M. pachydermatis* isolates from bears carry genotype B1, which is identical to one of the previously described genotypes from dogs (Puig et al., 2017), suggesting its transmission potential and adaptation to distantly related hosts. It cannot be ruled out that genotype B1 is even more widespread in other wild and domestic animals, but this requires further investigation. To address this question, it would be particularly interesting to examine the genetic variability of *M. pachydermatis* in populations of gray wolves, the closest wild relative of the dog. In contrast to the widely distributed B1 genotype, three other genotypes (B2-B4) isolated from five bears (approx. 10% of the samples Supplementary Table S1) were unique to bears. Although the B2 and B3 genotypes carry loci variants that have been identified in various domestic animals, the specific combinations found in bears have not been reported previously. In contrast, the B4 genotype consists of loci carrying bear-specific sequences. This may indicate that this particular *M. pachydermatis* genotype has emerged only recently and has not yet spread to other hosts. However, one cannot rule out the possibility that the B4 genotype has undergone evolutionary processes leading to the formation of a strain specifically adapted to bears.

In relation to body site sampling, it has been reported that the frequency of occurrence of some *Malasezzia* species depends on body site sampling (Cafarchia et al., 2008). In this study, most isolates sampled from different body sites of the same animal (left/right ear canal or anus) had identical genotypes. Only two out of 42 bears carried two *M. pachydermatis* genotypes (B1/B2 and B1/B4) at different body sites (Supplementary Table S1, bears 14 and 15). Although a rare event in our case, this is consistent with Guillot et al. (1997) who reported that the skin of an animal can be colonized by more than one type of *M. pachydermatis,* suggesting the possibility of multiple colonization events between individual animals.

**Figure 4:**
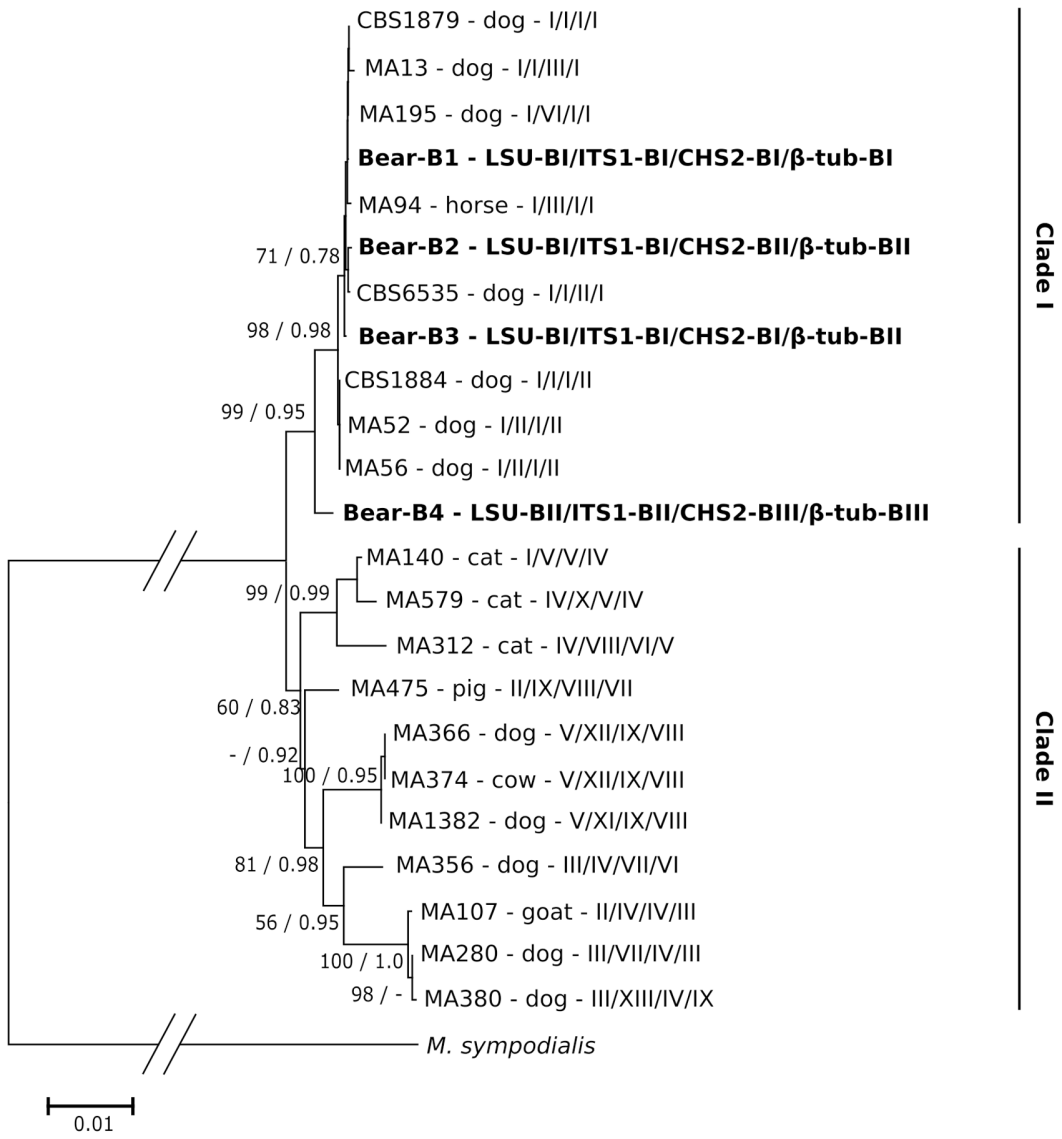
Maximum likelihood phylogenetic tree of *M. pachydermatis* genotypes combined of four gene loci (LSU/ITS1/CHS2/β-tub). The *M. pachydermatis* genotypes from brown bear host are shown in bold. Genotypes isolated from other host animals are marked as in Puig et al. (Puig et al., 2017); host animal as well as combination of respective gene loci are indicated. Numbers on nodes denote NJ bootstrap support/ML aLRT support (values lower than 50%/0.70 are not shown). Genotype of the respective gene loci of *M. sympodialis* was used as outgroup.

We also investigated the phylogenetic relationship of *M. pachydermatis* genotypes B1–B4 and those previously described in other animals. The concatenated matrix consisted of 2,228 aligned nucleotide positions (available in TreeBase). Phylogenetic analysis (Figure 4) revealed that *M. pachydermatis* genotypes B1–B4 from bears grouped in a well-supported (99%) clade I with several other genotypes (Puig et al., 2017) isolated mainly from dogs (i.e., only one from a horse). Three of the four genotypes of *M. pachydermatis* found in bears (B1-B3) are grouped close to some of the previously reported genotypes, while the much more divergent genotype B4 forms a separate branch within this clade, which is consistent with its loci sequence specificities. Clade II is moderately supported and consists of *M. pachydermatis* genotypes isolated from various domestic animals (cats, cows, pigs, goats, and dogs). The distances between genotypes within this clade are much higher and show several well supported subclades comprising predominantly *M. pachydermatis* genotypes from dogs, suggesting that dogs are the most common host for *M*. *pachydermatis* strains. In contrast, *M. pachydermatis* genotypes specific to cats form a well-supported subclade consisting of three quite variable genotypes, suggesting their common ancestry and subsequent independent evolution in this particular host. Similarly, the only genotype of *M. pachydermatis* so far recorded from pigs is distinctly different from all other genotypes, suggesting its evolutionary distance from other *M. pachydermatis* strains and its specialization to this particular host (Puig et al., 2017). The host specificity of *M. pachydermatis* has also been reported for some isolates obtained from rhinoceros, dogs and ferrets (Guillot et al., 1997).

As shown, phylogenetic analysis of concatenated sequence data (four loci) proves to be much more informative in the phylogenetic context and provides better resolution than the single locus approach. The results obtained demonstrate a close association between the genetic variants of *M. pachydermatis* found in bears and several of the numerous variants isolated from dogs.

It has been reported that *Malassezia* species can show distinct geographical differences (Velegraki et al., 2015). We reviewed a possible relationship between the different *M. pachydermatis* genotypes (B2-B4) and the geographic locations where bears were found. In this study we could not assign any genotype to a specific geographic location (Figure 1), which is most likely due to the long-distance migration routes of bears. Overall, genotype B1 is prevalent in bears, and most likely transmission to offspring occurs vertically, immediately after birth, as previously reported for dogs (Wagner and Schadler, 2000).

### The relationship between *Malassezia pachydermatis* genotypes with their MS spectra

**Figure 5:**
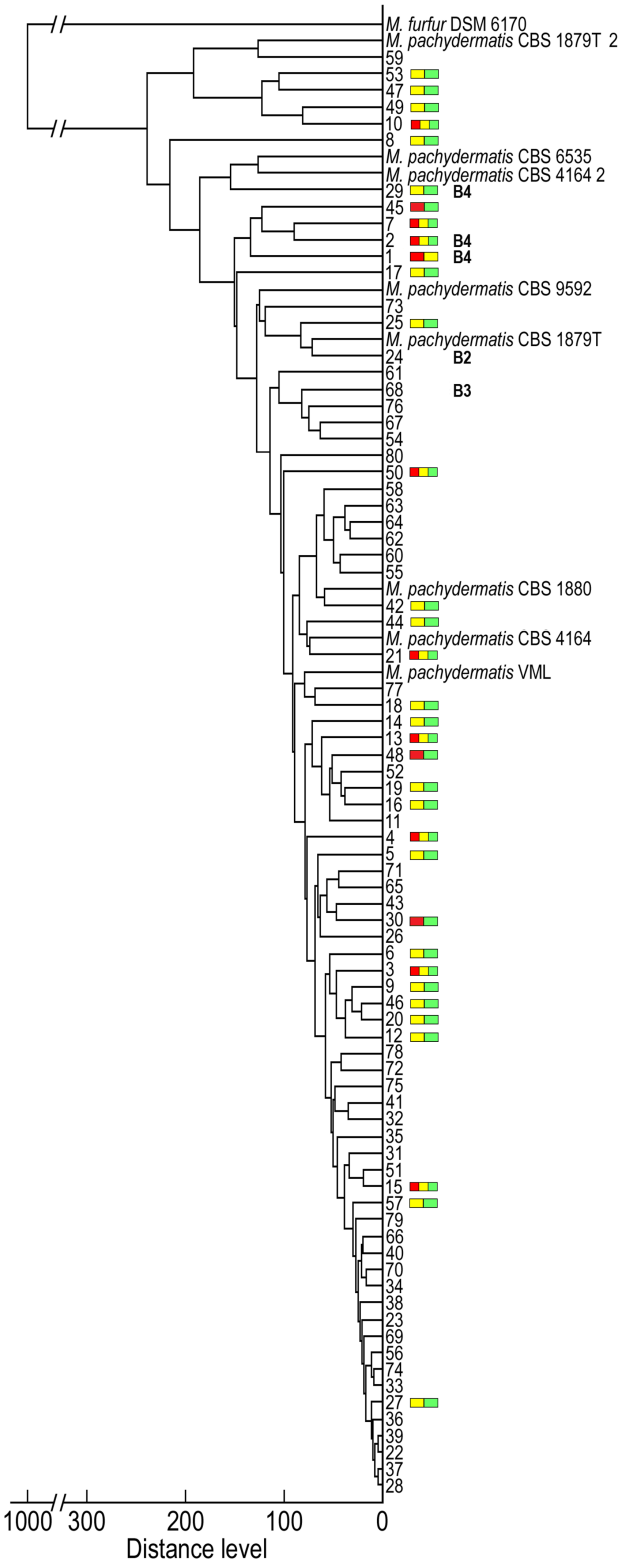
Dendrogram clustering the MALDI-TOF MSP obtained for *Malassezia* isolates using *M. pachydermatis* and one *M. furfur* referent spectra. Colored bars represent isolates that exhibit score fluctuation (Figure 2) whereas B2, B3 and B4, represents genotypes unique to yeast isolates from brown bears identified by molecular genotyping (all other numbers belong to isolates with B1 genotype).

Finally, we examined the relationships between the detected genotypes and their corresponding MS spectra. We performed an MSP cluster analysis of 80 *M. pachydermatis* isolates, in particular to gain a deeper insight into spectra variability of the identified unique bears’ genotypes (B2-B4) compared to B1. The score-oriented dendrogram of the isolates clustered based on their MSPs is shown in Figure 5. The isolates of *M. pachydermatis* are clearly separated from the congeneric species *M. furfur*. This dendrogram also shows the similarity of the isolates to the spectra of the reference strains of *M. pachydermatis* in the Bruker database.

As shown on the MSP dendrogram (Figure 5), the majority of isolates exhibited spectrum profiles with a Distance Level (DL) ≤ 100 and clustered with three *M. pachydermatis* reference strains (VML; CBS 4164 and CBS 1880). The others exhibited a slightly higher DL and either formed separate subgroups (e.g., isolates 53/47), branched separately (e.g., isolate 8), or grouped closer (e.g., isolate 24 or 29) to *M. pachydermatis* reference strain(s) (CBS 1879 T; CBS 9592; CBS 4164_2; CBS 6535 and CBS 1879T_2). With respect to the relationship between the identified genotypes and the spectrum profiles, isolates with the B1 genotype formed clades with the widest range of DLs. In contrast, bear-unique genotypes had only higher DLs, but these genotypes were dispersed into several branching subgroups, among which the B4 genotypes were clustered closer to each other and had slightly higher DL compared to B2 and B3. The 42.5% of isolates that had higher fluctuation in MALDI Biotyper scores (Figure 2) were present in all groups and were not evenly distributed across the dendrogram (Figure 5). Closer inspection reveals that most of them branch separately (e.g., 8, 29, 45, 1, 17, etc.) or are grouped with isolates that also exhibit higher fluctuation in scores (e.g., 53/47, 49/10, 7/2, 19/16, etc.). Species reliable scores were obtained using younger cell biomass for almost all of these isolates (33/34), which could be ascribed to some morphological changes during their growth. Indeed, cell wall thickness may be related to growth conditions and age of the colony (Guého-Kellermann et al., 2010). Consequently, our results could be explained by the fact that the proper lysis of the cells during the extraction process was partially impaired by a thicker cell wall and/or the colony texture of the isolates, which showed a higher fluctuation of the score values.

### MALDI-TOF MS shows protein profile relatedness among *Malassezia pachydermatis* isolates

**Figure 6:**
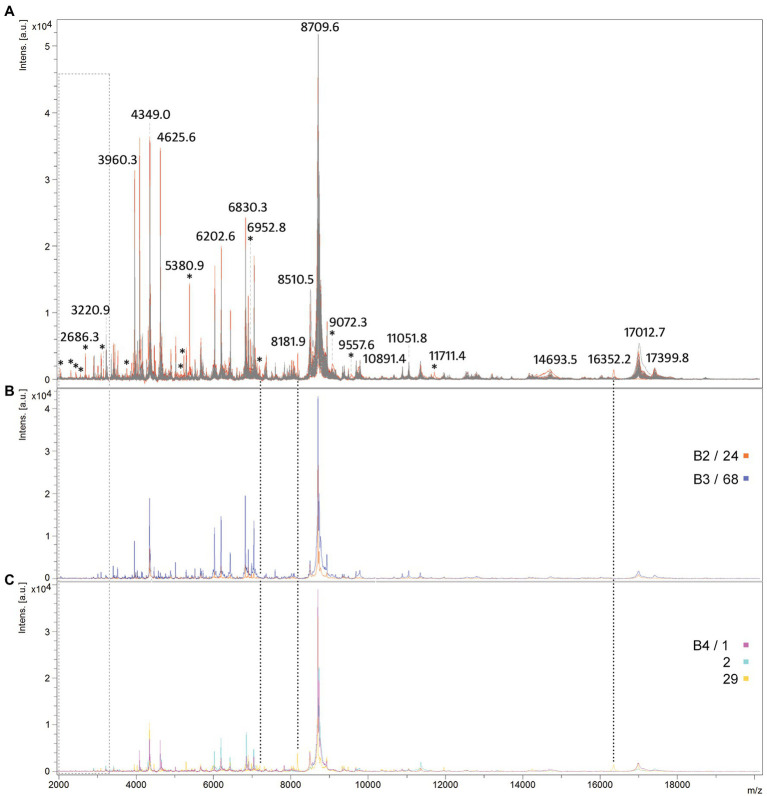
Comparison of protein spectra profiles of *M. pachydermatis* isolates. An overlaid view of mass spectra profiles **(A)** MS profiles of isolates exhibiting DL ≤ 100 are shown in gray while in red of isolates exhibiting DL > 100; **(B)** MS profiles of B2 and B3 genotypes; **(C)** MS profiles of B4 genotypes. Asterisks show all peaks unique to group exhibiting higher DL (red) while peaks uniquely produced by B4 genotype (sample 29) are marked with dotted lines and peaks that are absent from B2–B4 genotypes are marked with light gray rectangle.

The best MS profiles obtained for all isolates were overlaid to investigate protein profile relatedness between isolates. The protein spectra of two main groups subdivided on the arbitrary DL (those with DL ≤ 100 and with DL > 100) were marked in gray and red, respectively, to investigate whether there are some specific differences between them (Figure 6A). Comparison of the intensity and position of the m/z values of the spectra showed that the highest number of peaks is present in all *M. pachydermatis* isolates. However, a few peaks of higher intensity (e.g., 5,380 Da and 2,686 Da) and a few of lower intensity (< 2,500 Da, represented by four asterisks) were obtained only in isolates with higher DL (DL > 100). Interestingly, *M. pachydermatis* genotypes specific to bears (Figures 6B,C) produced spectra lacking peaks of lower molecular mass. Although the spectra of genotypes B2 and B3 produced higher intensity peaks, genotype B4 produced some unique peaks that were not found in other isolates. Overall, the use of MALDI-TOF MS showed significant potential to distinguish spectra between the two main subgroups of *M. pachydermatis* and also discovered some very specific peaks in genotype B4. Thus, the method proved to be a very rapid and powerful tool for the identification and subtle differentiation of *M. pachydermatis* genetic variants isolated from bears.

To the best of our knowledge, this is the first study to provide detailed insight into the genetic variability that has led to the identification of specific *M. pachydermatis* genotypes from wild. MALDI-TOF MS approach with adjustment of culturing conditions or by lowering the cut-off value provides rapid and reliable species identification. In addition, it exhibits a higher capacity to discriminate yeast variants. Overall, the results of this study indicate that *M. pachydermatis* has significant potential to be transmitted between distantly related hosts and its ability to adapt to different ecological niches.

## Data availability statement

The sequences and datasets presented in this study can be found in online repositories NCBI GenBank (accession numbers OQ519261, OQ519262, OQ519274, OQ519275, OQ550025-OQ550030) and TreeBase (http://purl.org/phylo/treebase/phylows/study/TB2:S29914).

## Ethics statement

The animal study was reviewed and approved by Committee of Veterinary Ethics, the Faculty of Veterinary Medicine, University of Zagreb, the Ministry of Agriculture, and the Ministry of Environmental and Nature Protection, Republic of Croatia.

## Author contributions

SH and DV conceived the research. LP, ĐH, SK, and DV provided project resources. SR and SH carried out sample collection and preservation. BBM, SH, SK, TP, and DV designed and performed the experiments. SH, SK, BBM, and DV analyzed and interpreted the data and wrote the manuscript, with input from TP and SR. All authors carefully reviewed the manuscript.

## Funding

This work was supported by the LIFE DINALP BEAR project (LIFE13 NAT/SI/000550), by Ministry of Science and Education of Croatia through the Program funding for academic years 2019–2022, by the Bernd Thies Foundation and Euronatur during all years of this project, and by the Croatian Government and the European Union through the European Regional Development Fund–the Competitiveness and Cohesion Operational Programme: project (KK.01.1.1.01.0002) granted to The Scientific Centre of Excellence for Bioprospecting–BioProCro and project “RAPTOVAX” (K.K.01.1.1.04.0099).

## Conflict of interest

The authors declare that the research was conducted in the absence of any commercial or financial relationships that could be construed as a potential conflict of interest.

## Publisher’s note

All claims expressed in this article are solely those of the authors and do not necessarily represent those of their affiliated organizations, or those of the publisher, the editors and the reviewers. Any product that may be evaluated in this article, or claim that may be made by its manufacturer, is not guaranteed or endorsed by the publisher.

## Footnotes

^1^http://purl.org/phylo/treebase/phylows/study/TB2:S29914

^2^http://www.atgc-montpellier.fr/phyml/

## References

[ref1] Aizawa T. Kano R. Nakamura Y. Watanabe S. Hasegawa A. (2001). The genetic diversity of clinical isolates of *Malassezia pachydermatis* from dogs and cats. Med. Mycol. 39, 329–334. doi: 10.1080/mmy.39.4.329.334 11556762

[ref2] Al-Sweih N. Ahmad S. Joseph L. Khan S. Khan Z. (2014). *Malassezia pachydermatis* fungemia in a preterm neonate resistant to fluconazole and flucytosine. Med. Mycol. Case Rep. 5, 9–11. doi: 10.1016/j.mmcr.2014.04.004, PMID: 24936403 PMC4052354

[ref3] Amend A. (2014). From dandruff to deep-sea vents: Malassezia-like fungi are ecologically hyper-diverse. PLoS Pathog. 10:e1004277. doi: 10.1371/journal.ppat.1004277, PMID: 25144294 PMC4140847

[ref4] Byrd A. L. Belkaid Y. Segre J. A. (2018). The human skin microbiome. Nat. Rev. Microbiol. 16, 143–155. doi: 10.1038/nrmicro.2017.157 29332945

[ref5] Cabanes F. J. (2014). Malassezia yeasts: how many species infect humans and animals? PLoS Pathog. 10:e1003892. doi: 10.1371/journal.ppat.1003892, PMID: 24586146 PMC3937305

[ref6] Cabañes F. J. Hernández J. J. Castellá G. (2005). Molecular analysis of Malassezia sympodialis-related strains from domestic animals. J. Clin. Microbiol. 43, 277–283. doi: 10.1128/JCM.43.1.277-283.2005, PMID: 15634983 PMC540166

[ref7] Cabañes F. J. Theelen B. Castellá G. Boekhout T. (2007). Two new lipid-dependent Malassezia species from domestic animals. FEMS Yeast Res. 7, 1064–1076. doi: 10.1111/j.1567-1364.2007.00217.x, PMID: 17367513

[ref8] Cafarchia C. Gasser R. B. Latrofa M. S. Parisi A. Campbell B. E. Otranto D. (2008). Genetic variants of *Malassezia pachydermatis* from canine skin: body distribution and phospholipase activity. FEMS Yeast Res. 8, 451–459. doi: 10.1111/j.1567-1364.2008.00358.x, PMID: 18294200

[ref9] Cafarchia C. Latrofa M. S. Figueredo L. A. Da Silva Machado M. L. Ferreiro L. Guillot J.. (2011). Physiological and molecular characterization of atypical lipid-dependent Malassezia yeasts from a dog with skin lesions: adaptation to a new host? Med. Mycol. 49, 365–374. doi: 10.3109/13693786.2010.531487, PMID: 21070187

[ref10] Cafarchia C. Stefania Latrofa M. Testini G. Parisi A. Guillot J. Gasser R. B.. (2007). Molecular characterization of Malassezia isolates from dogs using three distinct genetic markers in nuclear DNA. Mol. Cell. Probes 21, 229–238. doi: 10.1016/j.mcp.2007.01.002, PMID: 17320347

[ref11] Castella G. Coutinho S. D. Cabanes F. J. (2014). Phylogenetic relationships of Malassezia species based on multilocus sequence analysis. Med. Mycol. 52, 99–105. doi: 10.3109/13693786.2013.815372, PMID: 23902157

[ref12] Castella G. De Bellis F. Bond R. Cabanes F. J. (2011). Molecular characterization of Malassezia nana isolates from cats. Vet. Microbiol. 148, 363–367. doi: 10.1016/j.vetmic.2010.09.021, PMID: 20961713

[ref13] Chang H. J. Miller H. L. Watkins N. Arduino M. J. Ashford D. A. Midgley G.. (1998). An epidemic of *Malassezia pachydermatis* in an intensive care nursery associated with colonization of health care workers' pet dogs. N. Engl. J. Med. 338, 706–711. doi: 10.1056/NEJM199803123381102, PMID: 9494146

[ref14] Cho O. Sugita T. (2016). Low DNA sequence diversity of the intergenic spacer 1 region in the human skin commensal fungi *Malassezia sympodialis* and *M. dermatis* isolated from patients with Malassezia-associated skin diseases and healthy subjects. Mycopathologia 181, 839–842. doi: 10.1007/s11046-016-0034-3, PMID: 27371104

[ref15] Chow N. A. Chinn R. Pong A. Schultz K. Kim J. Gade L.. (2020). Use of whole-genome sequencing to detect an outbreak of *Malassezia pachydermatis* infection and colonization in a neonatal intensive care unit-California, 2015-2016. Infect. Control Hosp. Epidemiol. 41, 851–853. doi: 10.1017/ice.2020.73, PMID: 32370815

[ref16] Chryssanthou E. Broberger U. Petrini B. (2001). *Malassezia pachydermatis* fungaemia in a neonatal intensive care unit. Acta Paediatr. 90, 323–327. doi: 10.1111/j.1651-2227.2001.tb00312.x, PMID: 11332175

[ref17] Coutinho S. D. A. Sacristán C. Bueno M. G. Marigo J. Pissinatti A. Kierulff M. C.. (2020). *Malassezia japonica* is part of the cutaneous microbiome of free-ranging golden-headed lion tamarins (Leontopithecus chrysomelas - Kuhl, 1820). Med. Mycol. 58, 133–136. doi: 10.1093/mmy/myz017, PMID: 31220312

[ref18] Dall’ Acqua Coutinho S. Fedullo J. D. Corrêa S. H. (2006). Isolation of *Malassezia* spp. from cerumen of wild felids. Med. Mycol. 44, 383–387. doi: 10.1080/13693780500411006, PMID: 16772235

[ref19] Darriba D. Taboada G. L. Doallo R. Posada D. (2012). jModelTest 2: more models, new heuristics and parallel computing. Nat. Methods 9:772. doi: 10.1038/nmeth.2109, PMID: 22847109 PMC4594756

[ref20] Denis J. Machouart M. Morio F. Sabou M. Kauffmann-Lacroix C. Contet-Audonneau N.. (2017). Performance of matrix-assisted laser desorption ionization-time of flight mass spectrometry for identifying clinical Malassezia isolates. J. Clin. Microbiol. 55, 90–96. doi: 10.1128/JCM.01763-16, PMID: 27795342 PMC5228266

[ref21] Eguchi-Coe Y. Valentine B. A. Gorman E. Villarroel A. (2011). Putative Malassezia dermatitis in six goats. Vet. Derm. 22, 497–501. doi: 10.1111/j.1365-3164.2011.00980.x, PMID: 21535256

[ref22] Fell J. W. Boekhout T. Fonseca A. Scorzetti G. Statzell-Tallman A. (2000). Biodiversity and systematics of basidiomycetous yeasts as determined by large-subunit rDNA D1/D2 domain sequence analysis. Int. J. Syst. Evol. Microbiol. 50, 1351–1371. doi: 10.1099/00207713-50-3-1351, PMID: 10843082

[ref23] Gaitanis G. Bassukas I. D. Velegraki A. (2009). The range of molecular methods for typing Malassezia. Curr. Opin. Infect. Dis. 22, 119–125. doi: 10.1097/qco.0b013e328324ed19, PMID: 19283910

[ref24] Gaitanis G. Magiatis P. Hantschke M. Bassukas I. D. Velegraki A. (2012). The Malassezia genus in skin and systemic diseases. Clin. Microbiol. Rev. 25, 106–141. doi: 10.1128/CMR.00021-11, PMID: 22232373 PMC3255962

[ref25] Gaitanis G. Robert V. Velegraki A. (2006). Verifiable single nucleotide polymorphisms of the internal transcribed spacer 2 region for the identification of 11 Malassezia species. J. Dermatol. Sci. 43, 214–217. doi: 10.1016/j.jdermsci.2006.03.013, PMID: 16797927

[ref26] Gandra R. F. Gambale W. de Cássia Garcia Simão R. da Silva Ruiz L. Durigon E. L. de Camargo L. M. A.. (2008). Malassezia spp. in acoustic meatus of bats (Molossus molossus) of the Amazon region. Brazil. Mycopathol. 165, 21–26. doi: 10.1007/s11046-007-9079-7, PMID: 18046623

[ref27] Gao Z. Perez-Perez G. I. Chen Y. Blaser M. J. (2010). Quantitation of major human cutaneous bacterial and fungal populations. J. Clin. Microbiol. 48, 3575–3581. doi: 10.1128/JCM.00597-10, PMID: 20702672 PMC2953113

[ref28] Gemmer C. M. Deangelis Y. M. Theelen B. Boekhout T. Dawson T. L. Jr. (2002). Fast, noninvasive method for molecular detection and differentiation of Malassezia yeast species on human skin and application of the method to dandruff microbiology. J. Clin. Microbiol. 40, 3350–3357. doi: 10.1128/jcm.40.9.3350-3357.2002, PMID: 12202578 PMC130704

[ref29] Guého-Kellermann E. Boekhout T. Begerow D. (2010). “Biodiversity, phylogeny and ultrastructure” in Malassezia and the skin: Science and clinical practice. eds. Boekhout T. Mayser P. Guého-Kellermann E. Velegraki A. (Berlin, Heidelberg: Springer Berlin Heidelberg), 17–63.

[ref30] Guillot J. Bond R. (2020). Malassezia yeasts in veterinary dermatology: an updated overview. Fron. Cell Infect. Microbiol. 10:79. doi: 10.3389/fcimb.2020.00079, PMID: 32181160 PMC7059102

[ref31] Guillot J. Guého E. Chévrier G. Chermette R. (1997). Epidemiological analysis of *Malassezia pachydermatis* isolates by partial sequencing of the large subunit ribosomal RNA. Res. Vet. Sci. 62, 22–25. doi: 10.1016/s0034-5288(97)90174-0, PMID: 9160419

[ref32] Guindon S. Dufayard J.-F. Lefort V. Anisimova M. Hordijk W. Gascuel O. (2010). New algorithms and methods to estimate maximum-likelihood phylogenies: assessing the performance of PhyML 3.0. Syst. Biol. 59, 307–321. doi: 10.1093/sysbio/syq010, PMID: 20525638

[ref33] Gupta A. K. Boekhout T. Theelen B. Summerbell R. Batra R. (2004). Identification and typing of Malassezia species by amplified fragment length polymorphism and sequence analyses of the internal transcribed spacer and large-subunit regions of ribosomal DNA. J. Clin. Microbiol. 42, 4253–4260. doi: 10.1128/JCM.42.9.4253-4260.2004, PMID: 15365020 PMC516278

[ref34] Hall T. A. (1999). BioEdit: a user-friendly biological sequence alignment editor and analysis program for windows 95/98/NT. Nucl. Acids Symp. Ser. 41, 95–98.

[ref35] Honnavar P. Ghosh A. K. Paul S. Shankarnarayan S. A. Singh P. Dogra S.. (2018). Identification of Malassezia species by MALDI-TOF MS after expansion of database. Diagn. Microbiol. Infect. Dis. 92, 118–123. doi: 10.1016/j.diagmicrobio.2018.05.015, PMID: 30025965

[ref36] Huber Đ. Jakšić Z. Frković A. Štahan Ž. Kusak J. Majnarić D.. (2008). "Brown bear management plan for the Republic of Croatia". (ed.) Jakšić Z.. Ministry of Regional Development, Forestry and Water Management, Directorate for Hunting Ministry of Culture, Directorate for the Protection of Nature).

[ref37] Ilahi A. Hadrich I. Goudjil S. Kongolo G. Chazal C. Léké A.. (2018). Molecular epidemiology of a *Malassezia pachydermatis* neonatal unit outbreak. Med. Mycol. 56, 69–77. doi: 10.1093/mmy/myx022, PMID: 28371911

[ref38] Katoh K. Standley D. M. (2013). MAFFT multiple sequence alignment software version 7: improvements in performance and usability. Mol. Biol. Evol. 30, 772–780. doi: 10.1093/molbev/mst010, PMID: 23329690 PMC3603318

[ref39] Kearse M. Moir R. Wilson A. Stones-Havas S. Cheung M. Sturrock S.. (2012). Geneious basic: an integrated and extendable desktop software platform for the organization and analysis of sequence data. Bioinformatics 28, 1647–1649. doi: 10.1093/bioinformatics/bts199, PMID: 22543367 PMC3371832

[ref40] Kolecka A. Khayhan K. Arabatzis M. Velegraki A. Kostrzewa M. Andersson A.. (2014). Efficient identification of Malassezia yeasts by matrix-assisted laser desorption ionization-time of flight mass spectrometry (MALDI-TOF MS). Br. J. Dermatol. 170, 332–341. doi: 10.1111/bjd.12680 24125026

[ref41] Kumar S. Stecher G. Tamura K. (2016). MEGA7: molecular evolutionary genetics analysis version 7.0 for bigger datasets. Mol. Biol. Evol. 33, 1870–1874. doi: 10.1093/molbev/msw054, PMID: 27004904 PMC8210823

[ref42] Lautenbach E. Nachamkin I. Schuster M. G. (1998). *Malassezia pachydermatis* infections. N. Engl. J. Med. 339, 270–271. doi: 10.1056/NEJM199807233390414 9687254

[ref43] Lee J. Cho Y. G. Kim D. S. Choi S. I. Lee H. S. (2019). First case of catheter-related *Malassezia pachydermatis* fungemia in an adult. Ann. Lab. Med. 39, 99–101. doi: 10.3343/alm.2019.39.1.99, PMID: 30215238 PMC6143468

[ref44] Lefort V. Longueville J. E. Gascuel O. (2017). SMS: smart model selection in PhyML. Mol. Biol. Evol. 34, 2422–2424. doi: 10.1093/molbev/msx149, PMID: 28472384 PMC5850602

[ref45] Lorch J. M. Palmer J. M. Vanderwolf K. J. Schmidt K. Z. Verant M. L. Weller T. J.. (2018). Malassezia vespertilionis sp. nov.: a new cold-tolerant species of yeast isolated from bats. Persoonia 41, 56–70. doi: 10.3767/persoonia.2018.41.04, PMID: 30728599 PMC6344816

[ref46] Makimura K. Tamura Y. Kudo M. Uchida K. Saito H. Yamaguchi H. (2000). Species identification and strain typing of Malassezia species stock strains and clinical isolates based on the DNA sequences of nuclear ribosomal internal transcribed spacer 1 regions. J. Med. Microbiol. 49, 29–35. doi: 10.1099/0022-1317-49-1-29, PMID: 10628823

[ref47] Morris D. O. (2005). *Malassezia pachydermatis* carriage in dog owners. Emerg. Infect. Dis. 11, 83–88. doi: 10.3201/eid1101.040882, PMID: 15705327 PMC3294355

[ref48] Patel R. (2019). A moldy application of MALDI: MALDI-ToF mass spectrometry for fungal identification. J. Fungi (Basel) 5:4. doi: 10.3390/jof5010004, PMID: 30609833 PMC6463175

[ref49] Pinter L. Anthony R. M. Glumac N. Hajsig D. Pogačnik M. Drobnič-Košorok M. (2002). Apparent cross-infection with a single strain of *Malassezia pachydermatis* on a pig farm. Acta Vet. Hung. 50, 151–156. doi: 10.1556/AVet.50.2002.2.3, PMID: 12113169

[ref50] Prohic A. Jovovic Sadikovic T. Krupalija-Fazlic M. Kuskunovic-Vlahovljak S. (2016). Malassezia species in healthy skin and in dermatological conditions. Int. J. Dermatol. 55, 494–504. doi: 10.1111/ijd.13116 26710919

[ref51] Puig L. Bragulat M. R. Castella G. Cabanes F. J. (2017). Characterization of the species *Malassezia pachydermatis* and re-evaluation of its lipid dependence using a synthetic agar medium. PLoS One 12:e0179148. doi: 10.1371/journal.pone.0179148, PMID: 28586389 PMC5460872

[ref52] Puig L. Bragulat M. R. Castella G. Cabanes F. J. (2018). Phenotypic and genetic diversity of Malassezia furfur from domestic and zoo animals. Med. Mycol. 56, 941–949. doi: 10.1093/mmy/myx140, PMID: 29294061

[ref53] Puig L. Castellá G. Cabañes F. J. (2016). Cryptic diversity of *Malassezia pachydermatis* from healthy and diseased domestic animals. Mycopathologia 181, 681–688. doi: 10.1007/s11046-016-0026-3, PMID: 27283291

[ref54] Ran Y. He X. Zhang H. Dai Y. Li L. Bulmer G. S. (2008). Seborrheic dermatitis flare in a Dutch male due to commensal Malassezia furfur overgrowth. Med. Mycol. 46, 611–614. doi: 10.1080/13693780802140931, PMID: 18608906

[ref55] Robert M. G. Cornet M. Hennebique A. Rasamoelina T. Caspar Y. Pondérand L.. (2021). MALDI-TOF MS in a medical mycology laboratory: on stage and backstage. Microorganisms 9:1283. doi: 10.3390/microorganisms9061283, PMID: 34204665 PMC8231132

[ref56] Roman J. Bagla P. Ren P. Blanton L. S. Berman M. A. (2016). *Malassezia pachydermatis* fungemia in an adult with multibacillary leprosy. Med. Mycol. Case Rep. 12, 1–3. doi: 10.1016/j.mmcr.2016.05.002, PMID: 27354932 PMC4910295

[ref57] Rosenvinge F. S. Dzajic E. Knudsen E. Malig S. Andersen L. B. Løvig A.. (2013). Performance of matrix-assisted laser desorption-time of flight mass spectrometry for identification of clinical yeast isolates. Mycoses 56, 229–235. doi: 10.1111/myc.12000, PMID: 22924975

[ref58] Shokri H. (2016). Occurrence and distribution of Malassezia species on skin and external ear canal of horses. Mycoses 59, 28–33. doi: 10.1111/myc.12430, PMID: 26549307

[ref59] Singhal N. Kumar M. Kanaujia P. K. Virdi J. S. (2015). MALDI-TOF mass spectrometry: an emerging technology for microbial identification and diagnosis. Front. Microbiol. 6:791. doi: 10.3389/fmicb.2015.00791, PMID: 26300860 PMC4525378

[ref60] Sugita T. Boekhout T. Velegraki A. Guillot J. Hađina S. Cabañes F. J. (2010). “Epidemiology of Malassezia-related skin diseases” in Malassezia and the skin: science and clinical practice. eds. Boekhout T. Mayser P. Guého-Kellermann E. Velegraki A. (Berlin, Heidelberg: Springer), 65–119.

[ref61] Teoh Z. Mortensen J. Schaffzin J. K. (2022). Invasive *Malassezia pachydermatis* infection in an 8-year-old child on lipid parenteral nutrition. Case Rep. Infect. Dis. 2022:8636582. doi: 10.1155/2022/8636582, PMID: 35096432 PMC8794696

[ref62] Theelen B. Cafarchia C. Gaitanis G. Bassukas I. D. Boekhout T. Dawson T. L. (2018). Malassezia ecology, pathophysiology, and treatment. Med. Mycol. 56, S10–S25. doi: 10.1093/mmy/myx134, PMID: 29538738

[ref63] Velegraki A. Cafarchia C. Gaitanis G. Iatta R. Boekhout T. (2015). Malassezia infections in humans and animals: pathophysiology, detection, and treatment. PLoS Pathog. 11:e1004523. doi: 10.1371/journal.ppat.1004523, PMID: 25569140 PMC4287564

[ref64] Vlek A. Kolecka A. Khayhan K. Theelen B. Groenewald M. Boel E.. (2014). Interlaboratory comparison of sample preparation methods, database expansions, and cutoff values for identification of yeasts by matrix-assisted laser desorption ionization-time of flight mass spectrometry using a yeast test panel. J. Clin. Microbiol. 52, 3023–3029. doi: 10.1128/JCM.00563-14, PMID: 24920782 PMC4136148

[ref65] Wagner R. Schadler S. (2000). Qualitative study of Malassezia species colonisation in young puppies. Vet. Rec. 147, 192–194. doi: 10.1136/vr.147.7.192, PMID: 10985463

